# User-Centered Development of a Mobile App to Assess the Quality of Life of Patients With Cancer: Iterative Investigation and Usability Testing

**DOI:** 10.2196/44985

**Published:** 2023-09-26

**Authors:** Chantal N L Beutter, Katharina Zeller, Uwe M Martens, Bettina Pfleiderer, Christian Fegeler

**Affiliations:** 1 MOLIT Institute gGmbH Heilbronn Germany; 2 Clinic for Radiology, University of Münster Münster Germany; 3 Internal Medicine III for Hematology, Oncology and Palliative Medicine SLK Clinics GmbH Heilbronn Germany; 4 University of Applied Science Heilbronn Heilbronn Germany

**Keywords:** quality of life, cancer, mHealth, mobile health, patient empowerment, user-centered design, user, user centered, design, physical well-being, well-being, mental health, monitoring, development, usability

## Abstract

**Background:**

The treatment for cancer can have a negative impact not only on physical well-being but also on mental health and the quality of life (QoL). Health apps enable the monitoring of different parameters, but to date, there are only few that support patients with cancer and none that focuses on the assessment of QoL. Furthermore, patients as stakeholders are often only integrated at the late stage of the development process, if at all.

**Objective:**

The aim of this research was to develop and evaluate a smartphone app (Lion-App) to enable patients with cancer to autonomously measure the QoL with an iterative, user-centered approach.

**Methods:**

Patients with cancer were involved in a 3-stage process from conceptualization to the point when the app was available on the tester’s private device. First, focus groups with members (N=21) of cancer support groups were conducted to understand their expectations and needs. Thereafter, individual tests were performed. After developing a prototype that incorporated findings from the focus groups, a second test cycle was conducted, followed by a beta test lasting 2 months. In our app, the QoL can be assessed via a patient diary and an integrated questionnaire. Through all stages, usability was evaluated using the modular extended version of the User Experience Questionnaire (UEQ+), including the calculation of a key performance indicator (KPI). If possible, the impact of sex on the results was evaluated. As part of the beta test, usage rates as well as age-dependent differences were also assessed.

**Results:**

A total of 21 participants took part in the initial 3 focus groups. In the subsequent usability testing (N=18), 17 (94%) participants rated their impression through the UEQ+, with a mean KPI of 2.12 (SD 0.64, range: –3 to 3). In the second usability test (N=14), the mean KPI increased to 2.28 (SD=0.49). In the beta test, the usage rate of 19 participants was evaluated, of whom 14 (74%) also answered the UEQ+ (mean KPI 1.78, SD 0.84). An influence of age on the number of questionnaire responses in Lion-App was observed, with a decrease in responses with increasing age (*P*=.02). Sex-dependent analyses were only possible for the first usability test and the beta test. The main adjustments based on user feedback were a restructuring of the diary as well as integration of a shorter questionnaire to assess the QoL.

**Conclusions:**

The iterative, user-centered approach for development and usability testing resulted in positive evaluations of Lion-App. Our app was rated as suitable for everyday use to monitor the QoL of patients with cancer. Initial results indicated that the sex and age of participants seem to play only a minor role.

## Introduction

### Quality of Life and Cancer

Cancer is a noncommunicable disease with high prevalence and is considered the most common cause of death in an aging and growing society [1,2]. In 2020, 19.3 million new cases, with approximately 10 million deaths, were estimated to occur worldwide [3]. Even though the survival rate after diagnosis, depending on the type of cancer, can be high, cancer treatment often has severe side effects [4,5]. Symptoms, such as fatigue and nausea, can decrease patients’ quality of life (QoL). According to the World Health Organization (WHO), the QoL is defined as “an individual’s perception of their position in life in the context of the culture and value systems in which they live and in relation to their goals, expectations, standards and concerns” [6]. In the context of diseases, the health-related QoL is used. This describes a multidimensional concept that focuses on the patient’s subjective perceptions about the effects of illness and the impact of treatment on their daily life, including the physical, psychological, and social burden on the patient [7]. Although the QoL has become increasingly important, there is no gold standard available to assess it [8-10]. The QoL is mainly measured via questionnaires, indices, or patient diaries. One of the most widely used questionnaires for assessing the QoL in oncology is the Core Quality-of-Life Questionnaire (QLQ-C30) of the European Organization for Research and Treatment of Cancer (EORTC) [11].

Improving the QoL has a major impact on the patient’s therapy: An increase in the QoL not only improves satisfaction with the treatment but also significantly influences compliance and outcomes in a positive way and may increase the survival rate accordingly [8,9,12]. Even though the positive impact of an increase in the QoL has already been shown, none of the identified projects as well as none of the papers analyzed in a review [13] published in 2018 about health promotion and disease management have focused on the patient’s QoL itself. These previous results were confirmed by Stark et al [14], in a review of publications between 2010 and 2020 on health promotion and prevention, who found few papers focusing on mental health and well-being in general. In most cases, the QoL is considered a secondary outcome but not the focus of the survey [15-19]. Even when the QoL is considered a key component, research mainly focuses on the current QoL of patients at a given point of time. In longitudinal studies, for example, patients’ QoL is only assessed every few months instead of daily or weekly [20-23]. Thus, there is a need for tools to continuously monitor patients’ QoL, which may be enabled using health apps.

### Health Apps

Since patients with cancer are often treated as outpatients, monitoring and symptom support are necessary. In this context, the keywords in patient-reported outcomes and patient-generated health data are becoming increasingly important. Data from patients may be used to measure the effectiveness of treatment, improve the physician-patient relationship, and concomitantly increase patient satisfaction and improve the QoL [24,25].

Still, these data can only be collected with close involvement of the patient. There are many new approaches to include patients in therapy: one of them is the use of health apps. Many studies have shown a positive impact of mobile health interventions on the well-being or outcome of patients with cancer (eg, by supporting the monitoring of symptoms or providing relevant information in the personal context of the disease and therapy) [16,18,24,26,27]. One of the key challenges in the use of smartphones is the influence of different factors, such as the previous experience of the users, the comprehensibility of the survey, and the preparation of the information for the users [28]. This is especially important when patients need to interact directly with the developed system. Despite this, users are often not involved in development until fundamental decisions about the architecture or basic processes have already been made. Maramba et al [29] revealed that only about a third of all publications that conducted usability testing have incorporated user feedback into further development [29].

To consider patients’ needs, expectations, and experiences, they should be integrated into the development stages before the start of the implementation in order to discuss and form processes in cooperation with them. Even though applying a user-centered design is a common method for doing so, there are still different approaches to how a user-centered development may be realized [30-32]. The aim of this research was to develop and evaluate a smartphone app (Lion-App) to enable patients with cancer to autonomously measure their QoL with an iterative, user-centered approach. The impact of sex and age on the development process and product was also assessed.

## Methods

### Study Design

#### App Development

The app developed in this research is called Lion-App, which enables a longitudinal survey of the QoL in oncology using a smartphone. As questionnaires have high objectivity, reliability, and validity [33], as well as high sensitivity to monitor changes over time, the app provided an assessment of the QoL through the EORTC QLQ-C30 in our research. Responses to the questionnaires could either be viewed as individual answer sheets or displayed in an evaluation over time. The evaluation of the app included a calculated total score of the questionnaire responses as well as the individual progressions of the subscales. Additionally, it is possible for users to document their well-being via an integrated patient diary.

The aim of the app is to help patients gain a better awareness of their personal QoL. The goal is to support them in self-management through different stages of their disease as this knowledge can be used to specifically promote the QoL in therapy or to identify and address limitations in long-term survivorship. Therefore, the application of the app is detached from clinical treatment but describes a solution for patients to assess their QoL independently at home. As the app is intended to be installed on the patient’s private device, it was developed in a user-centered way to reduce usability issues for better applicability.

#### User-Centered Design

**Figure 1:**
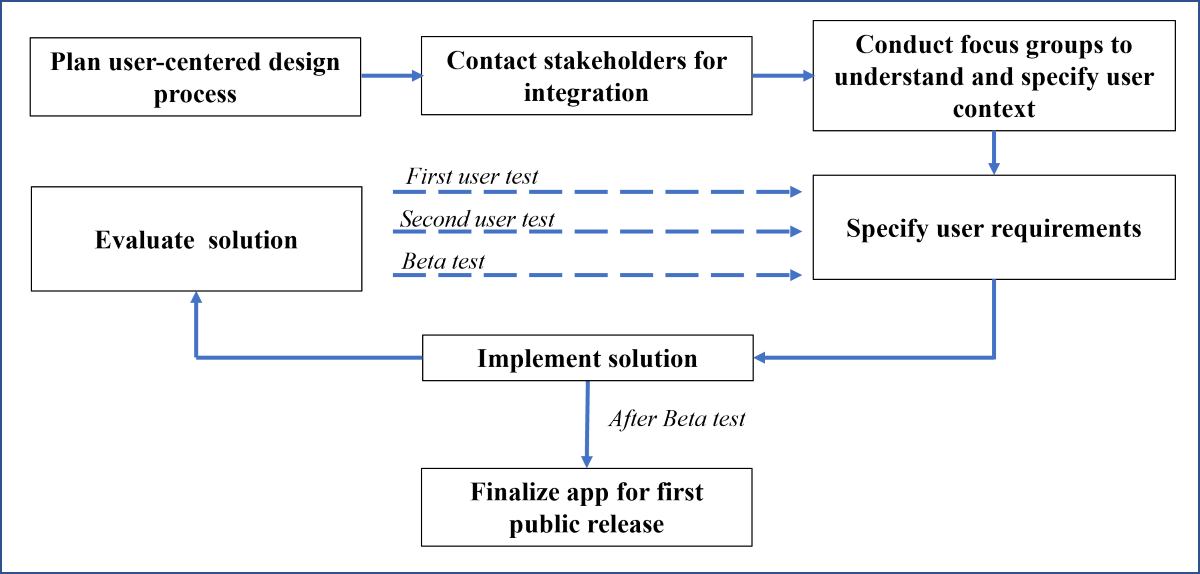
Process workflow of our user-centered design.

In general, the design of the app’s interfaces was based on the International Organization for Standardization (ISO) standard 9241, and a graphic designer managed the aspects of usability and user experience (UX). The goal was to develop an easy-to-use app for users to document their QoL through various functions. In doing so, the steps in the ISO 9241-210 process were performed iteratively, as seen in Figure 1. The first step in planning the human-centered design process was performed at the end of 2020. As a next step, several cancer support groups were contacted, and focus groups were conducted at the beginning of 2021 to understand and specify the user context. Consequently, user requirements were specified, which led to a first prototype for the first usability test (usability test 1) from April to June 2021. Based on the evaluation of this implementation, further design solutions were developed, which were again evaluated in October 2021 (usability test 2) and from December 2021 to February 2022 (beta test). In the process, the maturity of the implementation increased over 3 cycles (usability test 1, usability test 2, and beta test). Final adaptations in 2022 led to the release of the product afterward.

### Implementation

After the initial planning phase of the human-centered design process, several cancer support groups were contacted, to whom the project was briefly introduced and whose interest in participation was requested. The following cancer support groups provided consent to participate:

- Regional association of the support group for women in Baden-Württemberg/Bayern
- Regional association of the prostate cancer support group in Baden-Württemberg
- State association of patients with lung cancer and their relatives in Baden-Württemberg

Three focus groups were conducted for each support group. The aim of the focus groups was to collect the end users’ perceptions regarding eHealth apps and their needs to better understand user requirements. All focus groups were moderated, and a transcript writer was present.

The focus group for the support group for women in Baden-Württemberg was conducted on April 21, 2021 (n=8, 38%, all female), the focus group for the prostate cancer support group was conducted on June 8, 2021 (n=3, 14%, all male), and the focus group for the lung cancer support group was conducted on June 16, 2021 (n=10, 48%; n=6, 60%, female and n=4, 40%, male). At that time, legal restrictions due to the COVID-19 pandemic prohibited on-site meetings. Thus, all focus groups were performed online. Participants were free to participate in the meetings.

All focus groups were guide-oriented and structured in the same way: First, an introduction to Lion-App was provided. Second, questions about prior experiences with QoL surveys were elicited. Third, various options to map the QoL were presented and individual preferences evaluated, followed by discussions of their applicability and usefulness in a health app.

Finally, potential features of the app were presented and discussed. For an initial evaluation of the look of a possible color concept to be used in the app, an exemplary design of a dashboard was shown to the participants. This screen also included possible structural information about basic processes of the app (eg, how to access features from the start page). Participants were asked to rate this concept through a questionnaire. After they answered the questionnaire, the focus group concluded with an open discussion of additional suggestions. At the end of the meeting, participants were informed about individual usability tests, in which a more detailed insight into the app would be possible, and invited to participate in them. Patients included in the focus groups were re-invited to participate in all further test stages. In addition, new support group members participated, as well as nonmembers of any support group who learned about this project through word of mouth.

The basic user requirements and prioritization of functions derived from the focus groups were analyzed and used as a basis for the implementation of a first prototype. This prototype consisted of mockups that were visualized in Adobe XD. The first and second digital usability tests and the beta test (usability test 3) are described in more detail in Multimedia Appendix 1.

In short, through our development cycles, we moved step by step from a prototype in a controlled user environment to a real-world setting and release version of the app. In usability test 1, a prototype was presented based on mockups that were to be tested with predefined tasks. In usability test 2, the app was made available on a test device and users were asked to use the app to document their QoL without using predefined specific functions. In the final usability test (beta test), the app was installed on users’ private devices and there were no specifications for usage. Therefore, testing could be carried out to any extent. Afterward, the app was processed for public release. Written and verbal feedback was collected per test stage, analyzed, and processed for the next development steps.

Since the literature states that user engagement should only be measured if usability is already evaluated at a high level [26,34,35], we decided to assess user engagement only in the beta test after the previous usability tests. A combination of the passively collected usage pattern, subjective evaluations of the UX, and expectations was gathered for better insight. We also investigated whether the integration of certain elements for extrinsic or instinctive motivation (eg, certain gamification elements) would impact user engagement over a longer period of use.

### Evaluation

User evaluations of the usability of Lion-App were analyzed using verbal feedback by expressing the thoughts and expectations via the “thinking aloud” methodology during use of the app or at the end of usability tests or by observation of user behavior. These results were combined with the responses to the usability questionnaire that was included in all 3 usability tests.

To evaluate usability and the UX across our development stages, we decided to use different User Experience Questionnaires (UEQs) from the existing framework [36]. A short version of the general User Experience Questionnaire (UEQ-S) was used once in the focus groups. It enabled users to classify the answers provided into pragmatic quality (usability aspects), such as functionality or efficiency, and hedonic quality (satisfaction), such as innovativeness or novelty [37]. A modular extended version of the User Experience Questionnaire (UEQ+) was used for all further usability tests. This questionnaire can be modularly built from a list of different UX scales. We decided to use the scales assessing efficiency, clarity, intuitive use, usefulness, quality of content, and trustworthiness of content for our evaluation [38]. For each scale, the subjective importance can be rated. Both scale assessment and importance can be extracted with a 7-item Likert scale ranging from –3 to 3. The combination of these values can be used to calculate a key performance indicator (KPI) as well as the overall UX impression across evaluations [39]. Even though the assessment of the UX using the UEQ+ is not as common as when using other tools, such as the System Usability Scale (SUS), we decided on this approach because the UEQ+ scales provide information about potential gaps in performance. Other well-known survey methods, such as the SUS, often only provide information about whether usability problems exist and may need further work to identify the problems rather than relying on existing limitations.

To better classify the responses of participants, the UEQ+ was augmented by including additional questions about sex, age (assessed in 5-year increments), and previous experience with mobile devices. In usability test 2 as well as the beta test, participants were also asked to indicate whether they had previously participated in a usability test of Lion-App.

As the beta test was the first test conducted over a longer period and on participants’ private devices, we could additionally explore the impact of different displays and gamification elements on user engagement. For this, participants were randomly assigned to 3 versions of the app after registration. All versions had the same basic functionalities as version A but differed in add-ons, as shown in Table 1. We compared usage rates as well as the use of certain functions in detail, such as the number of responses provided to the integrated questionnaire, depending on the version and gender or age.

**Table 1:** Overview of the functionalities of the 3 versions of Lion-App used for the beta test.

Version	Functionalities	Add-ons
A	QLQ-C30^a^ Patient diary Information page for relevant topics related to cancer Push notifications as a reminder for usage	N/A^b^
B	QLQ-C30 Patient diary Information page for relevant topics related to cancer Push notifications as a reminder for usage	Count of active weeks^c^ Medals
C	QLQ-C30 Patient diary Information page for relevant topics related to cancer Push notifications as a reminder for usage	Extended evaluation: (1) comparison of scores and (2) display of IQR^d^

^a^QLQ-C30: Core Quality-of-Life Questionnaire. Users were able to fill out the questionnaire at any time. Questionnaire responses were visible as an answer sheet or in an evaluation (with a calculation of the scores).

^b^N/A: not applicable.

^c^Number of interactions≥1.

^d^Reference values from Scott et al [40].

Participants could only register for the beta test until 4 weeks before the end of the test cycle, as we determined that a minimum period of use of 4 weeks is required to evaluate usability and to be able to draw a conclusion about user engagement. Again, usability assessment was conducted using the UEQ+, and an additional questionnaire for the general impression and user engagement was administered. In this questionnaire, participants were able to provide an overall rating and state whether they would recommend the app and to what extent they could imagine themselves using it in the future. The results were also used to decide about the long-term integration of an extended evaluation or gamification elements.

Since participants had to answer 2 questionnaires during the beta test, the UEQ+ was assigned 2 weeks before the end of the test cycle, whereas the other questionnaire was administered after completion. After the beta test came closest to a real-world application of Lion-App, we decided not to conduct another test if the KPI was in the upper quarter (>1.5). In this case, feedback was still incorporated before the app was published.

### Participants

According to Nielsen and Landauer [41], 85% of usability problems can be identified with only 5 users and almost 100% can be identified with 15 users. Thus, we aimed to exceed the critical number of 5 users per usability test conducted and to reach ≥15 users, if possible.

Patients with cancer, during or after treatment of their cancer, and older than 18 years were included in the usability tests. The beta test contained 1 more exclusion criterion: as the app needed to be installed on a private mobile device, participants without a mobile device could not take part, as no one in the study could be provided with a smartphone. In addition, participants did not receive any compensation for participation. Throughout all test stages, interested parties were informed about further usability tests via support groups or email.

### Data Analysis

To assess user requirements, the transcripts of the respective focus groups were subsequently analyzed. This involved evaluating what interest and experience existed across participants and what prioritization of functions was preferred. Accordingly, the order of the functions to be implemented was determined. In addition to the KPI of the questionnaires of the UEQ framework, descriptive analyses of the mean score (SD), variance (var), confidence (C), and 95% CI were performed. For internal consistency of the scales, the Cronbach α coefficient was evaluated [42]. In addition, the median age of participants was calculated, and the Mann-Whitney U test was performed to detect differences between the UEQ+ ratings of the app based on sex. In the beta test, a *t* test for independent samples was additionally performed, including a test of equality of variance using the Levene test for the exploration of the effect of sex on the number and type of entries. For all age-dependent calculations, a 1-factor ANOVA was performed. ANOVA included all participants who indicated their age (12/19, 63%). Post hoc tests were only performed when the first analysis showed a significance of 5%. To determine the correlation with a 2-tailed significance of 5%, a Pearson correlation was performed. Before performing parametric tests, the normal distribution of data was assessed based on a significance value of >.05 in the Shapiro-Wilk test, which then was additionally confirmed with the corresponding Q-Q diagram as well as the histogram of the data.

SPSS version 28.0.1.1 (IBM Corp) was used for calculations. Detailed descriptive analyses of the UEQ-S and the overall UEQ+ are grouped in Multimedia Appendix 2, corresponding statistical calculations stratified by sex are summarized in Multimedia Appendix 3, and an overview of the interactions stratified by the app version of the beta test is provided in Multimedia Appendix 4.

### Ethical Considerations

According to the exclusion criteria of our local Münster German Ethic Kommission guidelines for the ethical evaluation of research projects of the University of Münster [43], this research did not need ethical approval, as participants were not exposed to any personal risk at any time and no individual personal data were evaluated: participants were exclusively considered as collective. The research did not include any interventions, nor did it influence the patients’ therapy. In addition, no health-related data were evaluated: participants only had to confirm to have received an oncological diagnosis in the past. Additionally, general conditions of the study, such as a review of the app development processes, were verified and approved by an external data protection officer. Data collection was exclusively performed in the context of evaluating usability and user engagement.

## Results

### Characteristics of Participants

Characteristics of the participants through the test stages are provided in Table 2. In general, no distinction was made between diagnoses, symptoms, or stages of disease, as the aim was to develop a generally applicable solution. Therefore, no additional clinical data were collected in addition to those shown in Table 2.

**Table 2:** Characteristics of participants through our stages of user-centered design. For the beta test, data could be drawn from the registration within the app as well as from the answers to the UEQ+^a^.

Characteristics			Focus group (N=21)		Usability test 1 (N=18)^b^		Usability test 2 (N=14)		Beta test (N=19; UEQ+ n=14, Lion-App: n=19)
Time period (months)			April-June 2021^c^		April-June 2021^c^		October 2021		December 2021-February 2022
**Sex, n (%)^d^**									
	Female	14 (67)		13 (72)		11 (79)		UEQ+: 8 (57); App: 10 (53)	
	Male	7 (33)		5 (28)		3 (21)		UEQ+: 6 (43); App: 9 (47)	
**Age (years), n (%)^d^**									
	30-34	N/C^e^		N/A^f^		1 (7)		UEQ+: N/A; App: N/A	
	40-44	N/C		N/A		N/A		UEQ+: N/A; App: 1 (5)	
	45-49	N/C		N/A		N/A		UEQ+: 1 (7); App: N/A	
	50-54	N/C		2 (12)		2 (14)		UEQ+: 3 (21); App: 2 (11)	
	55-59	N/C		4 (24)		4 (29)		UEQ+: 5 (36); App: 3 (16)	
	60-64	N/C		8 (47)		4 (29)		UEQ+: 1 (7); App: 2 (11)	
	65-69	N/C		1 (6)		1 (7)		UEQ+: 2 (14); App: 2 (11)	
	70-74	N/C		2 (12)		2 (14)		UEQ+: 2 (14); App: 1 (5)	
	75-79	N/C		N/A		N/A		UEQ+: N/A; App: 1 (5)	
	Not indicated	N/C		N/A		N/A		UEQ+: N/A; App: 7 (37)	
**Experience with mobile devices and apps, n (%)^d^**									
	Never worked with mobile devices	N/C		N/A		N/A		N/A	
	Rarely work with mobile devices	N/C		5 (30)		2 (14)		3 (21)	
	Often work with mobile devices	N/C		12 (70)		12 (86)		11 (79)	
**Participation in previous usability tests, n (%)^d^**									
	Yes	N/C		N/C		10 (71)		9 (64)	
	No	N/C		N/C		4 (29)		5 (36)	
**Version, n (%)**									
	A	N/C		N/C		N/C		9 (47)	
	B	N/C		N/C		N/C		6 (32)	
	C	N/C		N/C		N/C		4 (21)	

^a^UEQ+: extended version of the User Experience Questionnaire.

^b^One person was unable to complete the interview due to illness, so information in the UEQ+ and additional questions asked were based on 17 participants.

^c^Group-specific procedures (date of focus group), period of usability test 1: (1) regional association of the support group for women in Baden-Württemberg/Bayern (April 21, 2021), April-May 2021; (2) regional association of the prostatic cancer support group in Baden-Württemberg (June 8, 2021), June 2021; and (3) state association of patients with lung cancer and their relatives in Baden-Württemberg (June 16, 2021), June 2021.

^d^Unless otherwise stated, information was taken from the UEQ+.

^e^N/C: not collected.

^f^N/A: not applicable.

### Assessment of User Requirements

In general, most of the participants had no previous experience with the survey of the QoL but were interested in regular mapping of the QoL in their daily lives. In the discussion of the applicability of such an app, not only the app’s use in therapy but also the transition from treatment to posttreatment and survival was addressed. Within the discussion of the prioritization of features, documentation of the QoL through a patient diary and questionnaire was given the highest priority. Other aspects, such as automated recording of movement via sensors or the possibility of networking with others in the app, were perceived as positive but received lower prioritization.

Across all participants, the UEQ-S was completed 11 times. The evaluation indicated a higher pragmatic (mean 2.16, SD 0.76) than hedonic (mean 1.05, SD 1.43) quality. The confidence of the pragmatic quality was 0.45 (95% CI 1.71-2.61), whereas that of the hedonic quality was 0.85 (95% CI 0.21-1.90). Overall, without separating the qualities, a mean value of 1.64 (SD 0.73), with a confidence of 0.43 (95% CI 1.21-2.08) was achieved. Cronbach α was .91 across all participants.

As the UEQ-S evaluation achieved sufficient results, neither the basic process of accessing the main functions from the dashboard nor the color concept was adjusted for the first prototype.

### Usability Test 1

In total 18 participants underwent usability test 1. The test cycles for the support group for women were conducted from April 26 to May 7, 2021 (n=8, 44%, all female). In the same period, 2 (11%) additional people (nonmembers of any support group; n=1, 50%, male and n=1, 50%, female) were included after they asked to participate. Individual tests for the prostate cancer support group were conducted on June 6, 2021 (n=2, 11%, all male), and tests for the lung cancer support group were conducted from June 21 to July 1, 2021 (n=5, 28%; n=2, 40%, male and n=3, 60%, female). Each participant was given 30-45 minutes for the usability test. Within this period, participants were provided with a short introduction, tested the app, and evaluated their experience through the UEQ+. Since 1 (6%) participant from the support group for women canceled her participation before completing all tasks, the sum of UEQ+ responses was based on 17 (94%) of 18 participants.

**Figure 2:**
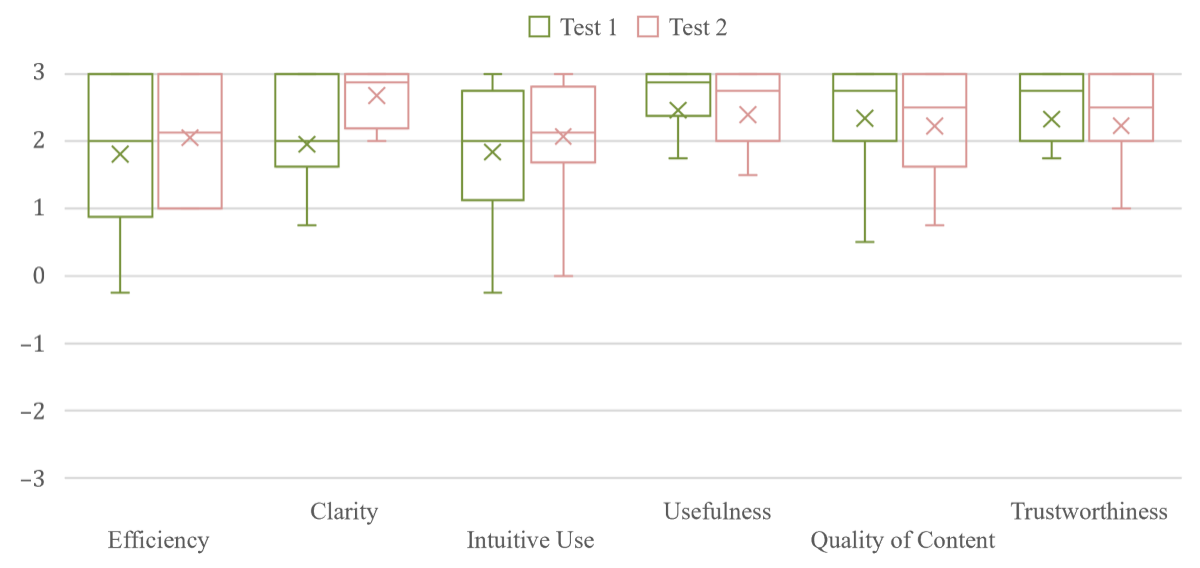
Overall rating of the UEQ+ (range: –3 to 3) for the first and second usability tests per scale. UEQ+: extended version of the User Experience Questionnaire.

All 18 (100%; median age 60 years) participants used a computer to open the dummy version of Lion-App in their preferred browser. A comparison of the assessment of the UEQ+ with the results of usability test 2 is displayed in Figure 2. Overall, a mean KPI of 2.12 (SD 0.64) was achieved, and Cronbach α was >.85 for all scales. Efficiency showed the lowest average (1.75) with the highest variance (1.75). Utility achieved the highest rating with a score of 2.43 and the lowest variance (0.89) in the survey.

A separate sex-dependent analysis of the rating was also carried out. The mean KPI of the female participants was 2.05 (SD 0.67) and that of the male participants was 2.24 (SD 0.57). The Mann-Whitney U test did not result in significant differences between sexes (*P*=.59).

The basic concept of the app was understood by most of the participants. The most common problem was that scrolling was overlooked. However, when overcoming the problem, participants stated that they would scroll more intuitively on a smartphone and that they would not change the process. Such input was tested in usability test 2, when the app was available on a test device.

In addition, more user feedback within the app was requested, such as success messages when saving a diary entry or a questionnaire response. As a result, toasts were planned to be integrated for such messages. Additionally, a new subpage was planned with a tutorial for all functions within the app.

### Usability Test 2

Since the app was provided on a test device for the second stage of usability testing, on-site meetings were necessary. As the lung cancer support group could not participate in meetings on-site, the number of participating groups reduced. Several local groups of the support group for women in Baden-Württemberg/Bayern, the prostate cancer support group, and the 2 nonmembers of usability test 1 participated from September 29 to October 18, 2021 (N=14; n=11, 79%, female and n=3, 21%, male). Again, the test period per person was set from 30 to 45 minutes.

Although the critical number of 5 persons was exceeded, the overall target of ≥15 users per usability test was not reached. The median age of participants was 60 years. An overview of the evaluation of the UEQ+ can be found in Figure 2. Across all participants, a mean KPI of 2.28 (SD 0.49) was achieved. No significant differences between ratings from usability tests 1 and 2 could be found through the Mann-Whitney U test (*P*=.65).

Since the number of male participants was below the critical sum of 5 users, no further analysis stratified by sex was performed.

### Beta Test

The app could be downloaded and tested from December 8, 2021, to February 7, 2022, for a maximum usage of 60 days (7.5 weeks). The app was installed a maximum of 20 times on Android and 2 times on iOS smartphones. In total, 20 participants registered in Lion-App, and 19 (95%) had at least 1 interaction. For further analysis of the research questions, the age and sex of the participants were taken from the user registration in the app. Again, the median age of the participants was 60 years. Across all responses to the UEQ+, a mean KPI of 1.78 (SD 0.84) was calculated. For all subscales, Cronbach α was >.85, except for the efficiency subscale (Cronbach α=.61).

Overall, 73 (64%) of 115 interactions were made by women. Furthermore, 54 (74%) of 73 diary entries and 19 (45%) of 42 questionnaire responses were submitted by women. The mean KPI for women was 1.46 (SD 0.98) and that for men was 2.2 (SD 0.23). The Mann-Whitney U test showed no statistical significance between sexes (*P*=.18). In addition, no significant sex-dependent differences were found in the general usage rate (*P*=.30), the use of the diary function (*P*=.09), or the number of questionnaire responses (*P*=.47).

Regarding the analysis of the usage rate related to the participant’s age, no impact of age was found on overall usage (*P*=.11) as well as on creating diary entries (*P*=.26). However, we did find an effect of age on the number of questionnaire responses (ANOVA *P*=.04). Of the 19 participants in the beta test, 7 (37%) did not indicate their age, which left 12 (63%) participants for further analysis. A 1-factor ANOVA with them resulted in *P*=.02 (*F*_5_=7.3), indicating that there is a strong negative correlation between age and questionnaire responses with a 2-tailed significance of 5% (Pearson correlation coefficient=–0.67, *P*=.02). Thus, the higher the age of the participant, the fewer the questionnaire responses submitted. The estimation of the dedicated 95% CI according to the r/z transformation of Fisher resulted in a lower threshold of –0.9 and an upper threshold of 0.16.

Due to an error in the code, the randomized assignment of participants into the 3 versions (A, B, and C; Table 1) did not lead to the same group size. Due to the uneven distribution of participants in the 3 app versions, group comparisons could not be performed. Even though no statistical evaluations were possible, we were able to extract the direction for further development by considering the subjective experience submitted by participants in the final questionnaire (n=7, 37%) as well as the verbal and written feedback.

**Figure 3:**
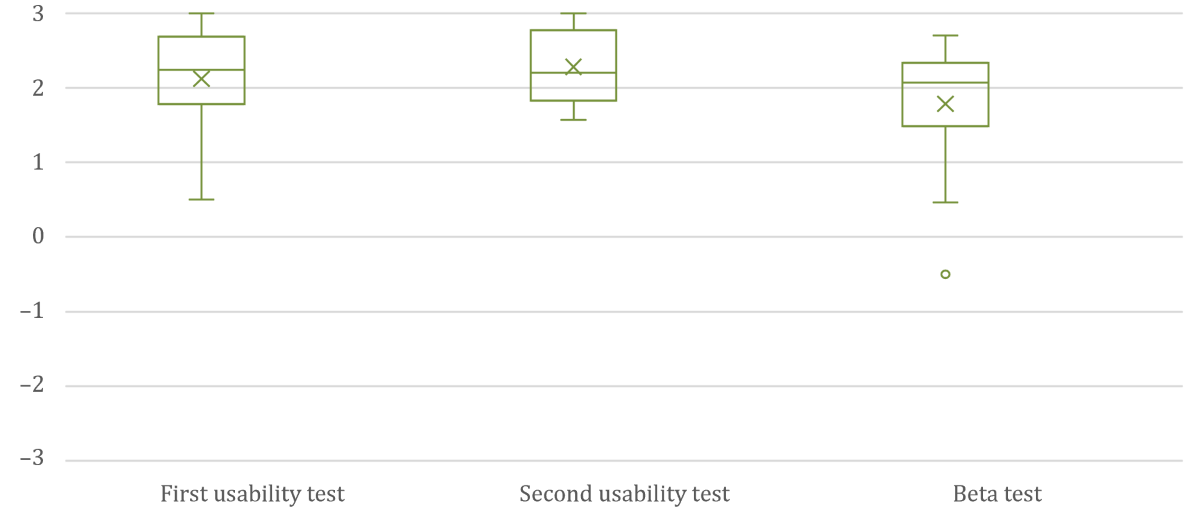
KPIs (range: –3 to 3) reflecting all three stages of usability testing. The KPIs are comparable in all 3 stages of development. KPI: key performance indicator.

Even though 4 (57%) of 7 answers were related to the basic version A without any add-ons, 5 (71%) of 7 users stated that they could imagine using the app on a daily-to-weekly basis in the future, and all respondents stated that they would recommend the app to others. Additionally, we analyzed the KPI through all test stages as the comparability of data was given as a minimum of 64% (n=9; see Table 2) of participants already engaged in previous usability tests. Figure 3 provides an overview of the trend. The results of the UEQ+ analysis across all stages as well as the evaluation of the app as applicable and recommendable in the beta test indicated that additional features of gamification and extended evaluation are not necessary for an app for a regular survey of the QoL. Therefore, we decided to exclude these features from further development. In addition to the finding that these additional features did not seem to have any influence, another conclusion could be drawn from the beta test: participants reported that the length of the QLQ-C30 had a negative impact on the regular use of the app. A regular response to 30 questions was too burdensome to be integrated into daily life, which led to the identification of a new user requirement for a shorter questionnaire. Furthermore, the beta test demonstrated that despite the extended explanation of how the title and category of the diary differed, problems continued to occur when using the diary. For this reason, the distinction between category and title was discarded after the beta test. We decided that an indication of a category would be deprecated but the input for a title would remain. To assist users in selecting an appropriate title, previous categories can be used to prefill the title as an auxiliary.

Since the measurement of the QoL worldwide does not seem to be influenced by sex or age [44], we decided to publish 1 general version in the Google Play Store and the Apple App Store after the KPI reached the specified minimum value of 1.5. Lion-App was released after incorporating the user requirements with regard to critical problems. Therefore, the input of a diary entry was adapted, and additionally, a shorter questionnaire, the EuroQol 5 Dimension 5 Level (EQ-5D-5L), for regular assessment of the QoL was integrated. As a requirement for further versions, personalized periods for push messages as a reminder for assessing the QoL were documented. In the beta test, the user requirement for reminders of usage from the second test cycle was also implemented through push messages. These were sent if users had been inactive for at least 7 days. In the evaluation of the beta version of the app, this feature was perceived as positive but a requirement to personalize this period could be identified.

## Discussion

### Principal Findings

An app for surveying the QoL of patients with cancer was developed, which was rated as acceptable and applicable by participants in the beta test following a user-centered development approach. The app has now been released in the Google Play Store and the Apple App Store. Even though there exist many health apps, few have been developed to support patients with cancer and none of them could be identified as focusing on assessment of the QoL. This is an important step toward increased patient empowerment of oncology patients in clinical settings by facilitating personalized treatment with closer inclusion of the patients’ QoL.

### Impact of Age and Sex

Even though no significant differences in the sex-dependent evaluation of the KPI were found, they cannot be ruled out completely, as for both analyses, from usability test 1 to the beta test, the average number of male testers was lower than that of female testers. In both evaluations, the SD was higher for women than for men. Interestingly, men rated the basic version of usability test 1 almost the same as the beta version (usability test 1=2.24, beta test=2.20), while the rating of the beta test by women was far below their rating of usability test 1 (usability test 1=2.05, beta test=1.46).

Due to the small number of men in usability test 1, there was most likely less variability in the evaluation data. Even though more men participated in the beta test, they used the app more seldom compared to women; 73 (64%) of 115 interactions were carried out by women. Therefore, we cannot rule out that the probability to detect a faulty function or a confusing process was driven by women, which might also be reflected in the KPI for the app’s rating. Moreover, as the research was conducted with a small cohort of 19 participants and within the environment of a beta test, sex differences of smaller effect sizes may have been overlooked and should be further assessed in an independent study.

Age was the only factor that influenced the entry form of a questionnaire response. Of note, the 95% CI of the correlation coefficient was 0, with a slightly higher upper threshold (0.16). This may indicate that there is no influence of age on the number of questionnaire responses within the app. However, with an upper value only slightly above 0 but the lower threshold almost at –1 (–0.9) and *P*=.02, the probability that there is an actual effect cannot be completely ruled out.

### Strengths and Limitations

One strength of our approach is that we not only focused on user-centered development but also included assessment of the QoL as a central component. Throughout our research, user-centered development led to good results on usability. A good acceptance of the concept over the development cycles was also demonstrated in the evaluation via the UEQ+: throughout all stages of development, the KPI of the UEQ+ responses was around 2. According to the literature, it is difficult to achieve a KPI above 2 with large cohorts, due to the different perceptions and experiences of the users [39].

Participants stated that they could imagine using the app for self-management in their daily lives. By including end users not only once at the beginning or end of a development but throughout the whole implementation, we were able to not only assess the expectations of users but also include their feedback directly in the concept and conduct testing of the revised implementation in the next iteration of usability tests. This enabled us to first identify expectations and problem areas and then focus on those functionalities step by step until a solution was found that is user friendly and matches user requirements.

Even though we achieved good evaluation results, our results might be biased as usability tests were conducted in cooperation with cancer support groups. The participants of such groups are already sensitive to questions related to the symptoms and treatment of their own disease. In addition, the “thinking aloud” methodology may cause participants to be more actively engaged with the app than they would normally be if they were using it in real life. This might also have influenced the outcomes of the tests. Furthermore, adding personal questions before the UEQ+ might have led to a bias in the evaluation.

Moreover, our study sample was not homogenous: patients with cancer during as well as after completion of therapy were included in our research. Neither the type of their cancer nor the year of diagnosis was controlled for. Moreover, as no medical records were collected or available, we had to rely on the participants’ self-reporting of having received a cancer diagnosis in the past. Another limitation may be the relatively low number of participants. When conducting usability tests with only small sample sizes, less deviations may occur within evaluations. Therefore, corroboration in a further study with a larger cohort to obtain robust results that could either confirm or reject identified trends is needed.

Still, regarding the evaluation of usability, the number of participants was sufficient according to the literature. On the one hand, the critical number of 5 participants by Nielsen and Landauer [41] was exceeded in all usability tests and the recommendation of the Common Industry Format (CIF) to include at least 8 people for usability research was met. In addition, Chomutare et al [45] recommended a number above 7 to be sufficient, and Spyridakis [46] reported that groups of 10-12 participants yield statistically significant results. In summary, our research exceeded these critical totals in all evaluations.

### Clinical Impact

The development of an app for the longitudinal survey of the QoL of patients with cancer provides the possibility of including the QoL and its monitoring into regular patient care. As data collection can be performed by patients at home, monitoring of the QoL can be performed easily. This provides the possibility of symptom-based treatment during therapy and assessment of the QoL posttherapy. By monitoring the QoL, not only can the quality of treatment be improved and better tailored to the patients’ needs but also the long-term impact of treatment may be better understood [47].

Studies have already shown a positive impact of symptom-based treatment on the course and outcome of therapy, but in clinical practice, assessing patients’ QoL is still challenging, since retrospective reports are usually distorted by recall bias [48]. It is therefore important to provide patients with a self-management tool that enables them to record their daily stress, symptoms, and QoL on a regular basis. Thus, not only the impact of treatment but also how it affects patients can be assessed [49]. The clinical impact of Lion-App has yet to be investigated.

### Conclusion

The results of our app development indicate that an iterative, user-centered approach leads to a solution that is both user friendly and can be used to document the patient’s QoL at home. This is an important step toward empowering and engaging patients to integrate them into therapy as part of the treatment team. Our results can serve as an example of how user-centered development may be executed. In this research, a standardized self-management tool for patients with cancer to assess their QoL was developed. As end users were integrated iteratively into all stages of the development process, this led to a continuous adaptation of user requirements, with improved usability to implement a solution tailored to the needs of end users. The results of this study may be used for scientific exchange on new approaches to the user-centered development of health apps and QoL research. In further research, the long-term applicability and acceptance of Lion-App should be assessed, as well as its clinical impact.

## Appendix

Multimedia Appendix 1 A detailed description of the procedure of user testing.

Multimedia Appendix 2 Detailed evaluations of the UEQ-S and UEQ+ of all usability tests. UEQ+: extended version of the User Experience Questionnaire; UEQ-S: short form of the User Experience Questionnaire.

Multimedia Appendix 3 Overview of sex-dependent evaluations.

Multimedia Appendix 4 Overview of interactions in the third usability test per version.

## Abbreviations

EORTC: European Organization for Research and Treatment of Cancer
KPI: key performance indicator
QLQ-C30: Core Quality-of-Life Questionnaire
QoL: quality of life
SUS: System Usability Scale
UEQ: User Experience Questionnaire
UEQ+: extended version of the User Experience Questionnaire
UEQ-S: short version of the User Experience Questionnaire
UX: user experience

## Data Availability

The data sets generated and analyzed during this study are not publicly available but may be requested from the corresponding author. These data sets only include data needed for the evaluation of usability and user engagement. In the research, no health-related data, such as quality-of-life data, were acquired.
