# Supplementary material for: User-Centered Development of a Mobile App to Assess the Quality of Life of Patients With Cancer: Iterative Investigation and Usability Testing
Source: JMIR Cancer. 2023 Sep 26;9:e44985. doi: 10.2196/44985 (PMC10565618; doi:10.2196/44985)
Supplement: Multimedia Appendix 1 [file cancer_v9i1e44985_app1.docx]

## Supplement A: Detailed description of the procedure of user testing

*Focus Groups:* User requirements were determined by three focus groups consisting of members from those three support groups who gave consent to participate. Depending on the number of questions, the introduction the app took between 27 and 40 minutes. The subsequent discussion of experiences and expectations ranged from 10 to 24 minutes, while the discussion of features took 9 to 18 minutes. Only after this discussion a first visualization was shown to the participants. Evaluating the dashboard via the UEQ-S took 4 to 9 minutes, while the open discussion at the end ranged from 4 to 13 minutes. Overall, the duration of the focus groups thus varied from 73 to 89 minutes.

*General procedure for the first and second user test sessions:* Interested individuals were able to sign up in advance at predetermined time slots. Users were then provided with information about the test, such as given tasks and/or instructions for a digital meeting in the first test. At the beginning of the test, users were informed about the test procedure, e.g. about the use of the ‘Think aloud” methodology to express their thoughts and expectations verbally. Afterwards, participants were given access to the prototype of the app (either online or on a test device). Users were instructed to work through various tasks chronologically and to indicate when a task was considered completed. Throughout the test session verbal feedback such as "this button confuses me," was documented as well as user behavior, like hesitation. Immediately after completing all tasks, participants were asked to evaluate the system anonymously through the UEQ+. In addition, further suggestions could be given written or verbally. In all tests a moderator and a transcript writer were present. All feedback interviews were guided, and the feedback allowed us to determine to what extend feedback given may be integrated into further versions of the app.

*First user test session:* Due to the ongoing COVID-19 pandemic, all test sessions were conducted online. Given tasks for the first user test included answering a questionnaire an editing an existing diary entry. While completing these tasks, test users were asked to share their screen. This allowed us to observe participants behavior and to analyze which elements of the interface they clicked. The session was not recorded at any time due to privacy reasons. As user tests were planned to take place shortly after the specification of user requirements through focus groups, an initial prototype was already tested by the women cancer support group in April 2021. This prototype was initially based only on the findings of the focus group of the women support group, as the focus groups of the prostate and lung cancer groups were not conducted until June 2021. The findings from these two subsequent focus groups were retrospectively compared with those of the women's support group. Adaptation of the prototype was then considered. Since no adjustment was necessary, the user tests of the prostate and lung support groups were conducted with the same prototype, allowing the comparability of results.

*Second user test session:* In the second user test the app was implemented on a smartphone. Tests were conducted on-site as the app was only available on a test device. Therefore, tests were carried out either at the premises of the cancer support groups or on-site at the MOLIT Institute. Tasks of the second user test were formulated more openly: Users were given a user story of a person who would like to use the app to document their QoL. Users were asked to test the functionality of the app and the possibilities of documentation of QoL without prior instructions. Additionally, users had to complete a registration within the app, as this process will be necessary later. As users had a free choice to what extent and through which features, they would assess their QoL it was documented which functions were preferably used. If users declared that they had completed the task and had only used limited features to document QoL, they were asked to test these missing functions as well. This aimed to have participants test all functions of the app. Through this we verified if the adaptation from the first user test led to a better usability and less uncertainty in the second version.

*Third user test session (beta test):* As a next step of our user centered development a beta test was carried out in the real world setting of the app. The beta test was conducted from December 2021 to February 2022 and differs not only in duration from the other two tests, but also in the context of examinations. Within the beta test, we explored the extent to which additional elements of gamification or evaluation influence user engagement. Additionally, it was explored whether the age or sex influence usage.
For participation, users were invited to install the app on their private device. To gain access to the application, users could register online for the beta test. At this registration, users were informed about planned evaluations throughout the test cycle. Before users could join the beta test they had to explicitly agree to the evaluation of their data. After registration, registered parties were equipped with a link to the Appstore to download the app. Here, the terms of use also stated that the app is currently published in a beta version which could lead to misfunctions and that anonymized analyses, e.g., of the usage rate, would be carried out. By installing the app, users agreed to those terms of use and therefore to the evaluations. If users did not install the app, no data was collected or evaluated. This concept of data evaluation and data protection was audited and approved by an external data protection officer. After installation, no instructions were given on how to use the app. Users were only asked to complete two evaluation questionnaires. Additionally, user behavior was analyzed. Participants were contacted by mail at the end of the beta test to thank them for their participation and to inform them about further development steps. After completing the beta test, all functions to enter data were deactivated as they were reviewed for revision. However, data that had already been entered could still be accessed.

*Summary:* Generally, after each test stage, the suggestions were noted and clustered to enable further development of the app. The results of the different test cycles were analyzed according to different criteria. On one hand, we looked at the possible consequences of the occurred usability problem. Here, different gradations were made, like a distinction between critical problems (e.g., user cannot use the function/app) and non-critical problems (e.g., user does not like the color). On the other hand, the frequency of occurrence of usage errors was also considered.
This way, a prototype consisting of mockups in Adobe XD could be improved to a release version step by step. From the findings of the first user test, the architecture for the implementation of a real application was decided and implemented. This led to the availability of the app on a test device for the second test cycle in October 2021. Again, the findings from the protected environment of a test device were processed to be used as preparation for the third and final user test (beta test), in which users could install the application on their private device for the first time and test it without any further instructions.
