# Supplementary material for: User-Centered Development of a Mobile App to Assess the Quality of Life of Patients With Cancer: Iterative Investigation and Usability Testing
Source: JMIR Cancer. 2023 Sep 26;9:e44985. doi: 10.2196/44985 (PMC10565618; doi:10.2196/44985)
Supplement: Multimedia Appendix 2 [file cancer_v9i1e44985_app2.docx]

## Supplement B: Detailed evaluations of the UEQ-S and UEQ+ of all user tests

Table B1: Overview of the results of the UEQ-S from the initial meeting: online focus group. (Var= Variance, SD= Standard deviation, C= Confidence, CI= Confidence Interval)

| **Item** | **Mean** | **Var** | **SD** | **N** | **C** | **CI** | | **Negative** | **Positive** | **Scale** |
| --- | --- | --- | --- | --- | --- | --- | --- | --- | --- | --- |
|  |  |  |  |  |  |  | |  |  |  |
| 1 | 2.2 | 0.4 | 0.63 | 11 | 0.37 | 1.30 | 2.57 | obstructive | supportive | Pragmatic Quality |
| 2 | 2.2 | 1.7 | 1.30 | 10 | 0.81 | 1.42 | 3.03 | complicated | easy | Pragmatic Quality |
| 3 | 2.0 | 0.4 | 0.67 | 11 | 0.39 | 1.61 | 2.39 | inefficient | efficient | Pragmatic Quality |
| 4 | 2.3 | 0.7 | 0.82 | 11 | 0.49 | 1.81 | 2.79 | confusing | clear | Pragmatic Quality |
| 5 | 0.9 | 2.3 | 1.52 | 11 | 0.90 | 0.00 | 1.80 | boring | exciting | Hedonic Quality |
| 6 | 1.4 | 1.8 | 1.35 | 11 | 0.80 | 0.60 | 2.20 | not interesting | interesting | Hedonic Quality |
| 7 | 0.3 | 2.5 | 1.58 | 10 | 0.98 | -0.65 | 1.31 | conventional | inventive | Hedonic Quality |
| 8 | 0.8 | 2.8 | 1.66 | 11 | 0.98 | -0.16 | 1.80 | usual | leading edge | Hedonic Quality |

Table B2: Overall results of the UEQ+ [-3,3] from test 1 (N=17). (Var= Variance, SD= Standard deviation, C= Confidence, CI= Confidence Interval)

| **Scale** | **Mean** | **Var** | **SD** | **N** | **C** | **CI** | | **Cronbach Alpha** |
| --- | --- | --- | --- | --- | --- | --- | --- | --- |
|  |  |  |  |  |  |  | |  |
| **Efficiency** | 1.75 | 1.75 | 1.31 | 17 | 0.62 | 1.13 | 2.37 | 0.87 |
| **Clarity** | 2.17 | 1.16 | 1.07 | 17 | 0.51 | 1.66 | 2.68 | 0.90 |
| **Intuitive Use** | 1.92 | 1.27 | 1.12 | 17 | 0.53 | 1.39 | 2.45 | 0.94 |
| **Usefulness** | 2.43 | 0.89 | 0.94 | 17 | 0.45 | 1.99 | 2.88 | 0.97 |
| **Quality of Content** | 2.27 | 1.04 | 1.01 | 17 | 0.48 | 1.79 | 2.76 | 0.96 |
| **Trustworthiness of Content** | 2.27 | 1.05 | 1.02 | 17 | 0.48 | 1.79 | 2.76 | 0.98 |

Table B3: Overall results of the UEQ+ [-3,3] from test 2 (N=14). (Var= Variance, SD= Standard deviation, C= Confidence, CI= Confidence Interval)

| **Scale** | **Mean** | **Var** | **SD** | **N** | **C** | **CI** | | **Cronbach Alpha** |
| --- | --- | --- | --- | --- | --- | --- | --- | --- |
|  |  |  |  |  |  |  | |  |
| **Efficiency** | 2.05 | 1.29 | 1.12 | 14 | 0.59 | 1.46 | 2.64 | 0.77 |
| **Clarity** | 2.68 | 0.26 | 0.50 | 14 | 0.26 | 2.41 | 2.94 | 0.85 |
| **Intuitive Use** | 2.07 | 0.76 | 0.86 | 14 | 0.45 | 1.62 | 2.52 | 0.95 |
| **Usefulness** | 2.39 | 0.90 | 0.94 | 14 | 0.49 | 1.90 | 2.88 | 0.96 |
| **Quality of Content** | 2.22 | 0.89 | 0.94 | 14 | 0.49 | 1.73 | 2.71 | 0.88 |
| **Trustworthiness of Content** | 2.18 | 0.82 | 0.90 | 14 | 0.47 | 1.71 | 2.65 | 0.97 |

Table B4: Overall results of the UEQ+ [-3,3] of the beta version of the app (N=14). (Var= Variance, SD= Standard deviation, C= Confidence, CI= Confidence Interval)

| **Scale** | **Mean** | **Var** | **SD** | **N** | **C** | **CI** | | **Cronbach Alpha** |
| --- | --- | --- | --- | --- | --- | --- | --- | --- |
|  |  |  |  |  |  |  | |  |
| **Efficiency** | 0.96 | 1.53 | 1.22 | 14 | 0.64 | 0.32 | 1.61 | 0.61 |
| **Clarity** | 1.82 | 0.95 | 0.97 | 14 | 0.51 | 1.32 | 2.33 | 0.93 |
| **Intuitive Use** | 1.68 | 2.44 | 1.55 | 14 | 0.81 | 0.87 | 2.49 | 0.90 |
| **Usefulness** | 1.98 | 1.07 | 1.03 | 14 | 0.54 | 1.44 | 2.52 | 0.93 |
| **Quality of Content** | 1.91 | 1.21 | 1.09 | 14 | 0.57 | 1.34 | 2.48 | 0.88 |
| **Trustworthiness of Content** | 2.30 | 0.72 | 0.84 | 14 | 0.44 | 1.86 | 2.75 | 0.85 |
