# Supplementary material for: User-Centered Development of a Mobile App to Assess the Quality of Life of Patients With Cancer: Iterative Investigation and Usability Testing
Source: JMIR Cancer. 2023 Sep 26;9:e44985. doi: 10.2196/44985 (PMC10565618; doi:10.2196/44985)
Supplement: Multimedia Appendix 3 [file cancer_v9i1e44985_app3.docx]

### Supplement C: Overview of sex dependent evaluations

Table C1: Overview of the ratings of the UEQ+ stratified by sex of the male (female) patients in test 1. (Var= Variance, SD= Standard deviation, C= Confidence, CI= Confidence Interval)

| **Scale** | **Mean** | **Var** | **SD** | **N** | **C** | **CI** | | **Cronbach Alpha** |
| --- | --- | --- | --- | --- | --- | --- | --- | --- |
|  |  |  |  |  |  |  | |  |
| **Efficiency** | 1.67 (1.80) | 2.32 (1.45) | 1.49 (1.19) | 6 (11) | 1.19 (0.70) | 0.47 (1.10) | 2.86 (2.50) | 0.97 (0.79) |
| **Clarity** | 2.65 (1.90) | 0.42 (1.39) | 0.63 (1.16) | 6 (11) | 0.51 (0.69) | 2.15 (1.21) | 3.16 (2.59) | 0.81 (0.90) |
| **Intuitive Use** | 1.96 (1.90) | 1.52 (1.15) | 1.21 (1.06) | 6 (11) | 0.97 (0.62) | 0.99 (1.27) | 2.92 (2.52) | 0.97 (0.91) |
| **Usefulness** | 2.05 (2.63) | 1.42 (0.55) | 1.16 (0.73) | 6 (11) | 0.93 (0.43) | 1.12 (2.19) | 2.98 (3.06) | 0.98 (0.97) |
| **Quality of Content** | 2.65 (2.03) | 0.24 (1.43) | 0.48 (1.18) | 6 (11) | 0.38 (0.70) | 2.27 (1.34) | 3.03 (2.73) | 0.94 (0.96) |
| **Trustworthiness of Content** | 2.65 (2.06) | 0.34 (1.35) | 0.57 (1.14) | 6 (11) | 0.46 (0.68) | 2.19 (1.38) | 3.11 (2.73) | 0.97 (0.98) |

Table C2: Overview of the ratings of the UEQ+ stratified by sex of the male (female) patients for the beta version of the app. (Var= Variance, SD= Standard deviation, C= Confidence, CI= Confidence Interval)

| **Scale** | **Mean** | **Var** | **SD** | **N** | **C** | **CI** | | **Cronbach**  **Alpha** |
| --- | --- | --- | --- | --- | --- | --- | --- | --- |
|  |  |  |  |  |  |  | |  |
| **Efficiency** | 1.42 (0.63) | 1.12 (1.6) | 1.04 (1.24) | 6 (8) | 0.83 (0.86) | 0.59 (-0.24) | 2.25 (1.49) | -0.06 (0.74) |
| **Clarity** | 2.08 (1.63) | 0.51 (1.21) | 0.70 (1.08) | 6 (8) | 0.56 (0.75) | 1.52 (0.87) | 2.65 (2.38) | 0.83 (0.97) |
| **Intuitive Use** | 2.50 (1.06) | 0.52 (3.03) | 0.71 (1.71) | 6 (8) | 0.57 (1.19) | 1.93 (-0.12) | 3.07 (2.25) | -3.59 (0.91) |
| **Usefulness** | 2.42 (1.66) | 0.43 (1.33) | 0.64 (1.13) | 6 (8) | 0.51 (0.79) | 1.90 (0.87) | 2.93 (2.44) | 0.79 (0.94) |
| **Quality of Content** | 2.33 (1.59) | 0.41 (1.25) | 0.62 (1.25) | 6 (8) | 0.50 (0.86) | 1.83 (0.73) | 2.83 (2.46) | -0.57 (0.91) |
| **Trustworthiness of Content** | 2.50 (2.16) | 0.35 (0.97) | 0.58 (0.97) | 6 (8) | 0.46 (0.67) | 2.04 (1.48) | 2.96 (2.83) | 0.63 (0.89) |
