# Supplementary material for: User-Centered Development of a Mobile App to Assess the Quality of Life of Patients With Cancer: Iterative Investigation and Usability Testing
Source: JMIR Cancer. 2023 Sep 26;9:e44985. doi: 10.2196/44985 (PMC10565618; doi:10.2196/44985)
Supplement: Multimedia Appendix 4 [file cancer_v9i1e44985_app4.docx]

## Supplement D: Overview of interactions within third user test per version

Table D1: Overview of the interactions within the app stratified by the assessed three app versions. Version A: Basic version without add-Ons; Version B: Basic version with gamification elements as add-On; Version C: Basic version with an extended evaluation as add-On.

| **Version and interaction form** | | **Frequency** | **Percentile** |
| --- | --- | --- | --- |
|  | |  |  |
| **A** | Diary entry | 42 | 67.7 |
|  | Questionnaire response | 20 | 32.3 |
|  | Total | 62 | 100.0 |
| **B** | Diary entry | 23 | 62.2 |
|  | Questionnaire response | 14 | 37.8 |
|  | Total | 37 | 100.0 |
| **C** | Diary entry | 8 | 50.0 |
|  | Questionnaire response | 8 | 50.0 |
|  | Total | 16 | 100.0 |
